# Bibliographical Notices

**Published:** 1853-07

**Authors:** 


### BIBLIOGRAPHICAL NOTICES.

*Elements of Chemistry; for the use of Colleges, Academies and Schools.* By M. V. REGNAULT. *Illustrated by nearly* 700 *wood cuts.* Translated from the French by T. FORREST BETTON, M. D., M. A. N. S., Fellow of the College of Physicians, Philada., &c., and Edited, with notes, by JAMES C. BOOTH, Melter and Refiner, U. S. Mint; and WILLIAM L. FABER, Metallurgist and Mining Engineer. Second Edition; Philada., Clark & Hesser; 1853. 2 vols. oct. pp. 1475.

In the last number we had occasion to speak of Löwig's Organic Chemistry. We now are called on to notice a chemical work of quite a different stamp. M. Regnault has for several years occupied a very favorable position among the scientific men of Paris, owing to his valuable investigations and discoveries in various of the higher departments of chemical research. His mind appears to have a practical direction, and in the work of which the above is the title page, he has exhibited this trait by bringing in numerous applications of his favorite science to the useful and ornamental arts and manufactures. In glancing over the pages, we perceive that the chemistry of the imponderables, usually occupying much space, has been omitted as a separate treatise, and that crystallography is largely dilated upon, with numerous excellent wood cuts, illustrating the several systems of crystals. Arsenic is placed among the non-metallic bodies at the side of phosphorus, a change of position which many chemists seem disposed to effect, although it is usually spoken of and described as a metal.

In the chapter preliminary to the metals, M. Regnault has introduced a short account of the geological structure of the earth's crust, with numerous cuts exhibiting stratification, metallic veins, etc. This is followed by a discussion of the general characters and formation of salts, in which the author takes the ground that those substances only should be considered salts which consist of two binary compounds, one of which acts as an electro-positive element or base, and the other in an electro-negative manner as an acid. Hence, he ejects the chlorides, iodides, etc. from the list. The solubility of salts is treated of in great detail, with a copper plate engraving, exhibiting the curves of solubility of the more important salts. The first class of metals includes those of the alkaline, alkaline earthy, and earthy bases; among the special notices we may mention those of the preparation of potassium, alkalimetry, nitrate of potassa and borate of soda; the manufacture, testing and analysis of gunpowder; building materials, cements and mortars; the glass manufacture and the several kinds of pottery. The metallurgy of the several important metals of the second class is introduced, and thoroughly illustrated with engravings. The steel and bronze manufacture, cannon casting, electro-plating and gilding, etc., are among the special notices.

The organic department is preceded by an elaborate account of proximate and ultimate organic analysis, the method of ascertaining formulæ, the plan of determining the acid, basic and neutral substances, and the methods, precautions and calculations necessary in ascertaining the specific gravity of the vapors and other physical properties of volatile organic bodies.

M. Regnault has not treated the chemistry of organic bodies so fully as the other department. His views appear unfavorable to the radical theory, yet they were published before the recent discoveries of Frankland and Kolbe, which have given so much probability to that view of organic constitution. He has not attempted a scientific classification;—commencing with the hydrates of carbon and their derivatives by the action of acids and ferments, and concluding with an account of animal chemistry and the principle manufactures of organic products, such as bread making, brewing, fermented liquors, the preparation of sugar from the beet and cane, of bone-black, charcoal, leather and illuminating gas.

The original work is in four duodecimo volumes; as now presented it is embraced in two large octavos of 700 and 800 pages. The beautiful wood cuts of the original are, in most instances, well copied, the letter press is large and distinct, and the paper good. Dr. Betton has given a fair and lucid translation, free from idiomatic peculiarities. The Editors have given proof of a careful revision. They have expressed the temperature by Fahrenheit's scale, and have adopted the hydrogen scale of equivalents, which will add much to its practical usefulness. On the other hand they have left the decimal weights and measures. The attempt at rendering these would have lost its value in the constant recurrence of fractions, as expressed in the English system.

The Editorial additions are in the form of foot notes, some of which are particularly useful. Mr. Faber has very properly given an exposé of the recent isolation of compound radicals; and among the notes of Prof. Booth we observe one on the process for refining of gold in the U. S. Mint.

On the whole, we cannot but deem the publication of Regnault's Chemistry a valuable addition to the chemical literature of the United States, as a book which should be within reach of every chemist, and calculated to be of essential service to artists and manufacturers.

*The Action of Medicines in the System; or "On the Mode in which Therapeutic Agents Introduced into the Stomach produce their Peculiar Effects on the Animal Economy."* By FREDERICK WILLIAM HEADLAND, B. A., M. R. C. S., &c. Philadelphia: Lindsay & Blakiston, 1853, pp. 560.

This monograph is a *Prize Essay*, "to which the Medical Society of London awarded the Fothergillian Gold Medal for MDCCCLII," and we feel little hesitation in saying, that it is the most thorough, masterly, and scientific exposition of the state of medical knowledge on the important topic treated, that has ever appeared. The subject to which the Essay was written is, "On the mode in which Therapeutic Agents introduced into the Stomach produce their peculiar effects on the animal economy." It is treated by the author in a series of distinct propositions, which he lays down and endeavors to demonstrate after the manner of mathematical theorems. We quote, as perhaps affording the best insight into the subject matter of the book, the author's series of *propositions* at length.

"*Prop.* I.—That the great majority of medicines must obtain entry into the blood, or internal fluids of the body, before their action can be manifested.

*Prop.* II.—That the great majority of medicines are capable of solution in the gastric or intestinal secretions, and pass without material change, by a process of absorption, through the coats of the stomach and intestines, to enter the capillaries of the portal system of veins.

*Prop.* III.—That those medicines which are completely insoluble in water, and in the gastric and intestinal juices, cannot gain entrance into the circulation.

*Prop.* IV.—That some few remedial agents act locally on the mucous surface, either before absorption, or without being absorbed at all. That they are chiefly as follows:

*a.* Irritant Emetics.

*b.* Stomach Anæsthetics.

*c.* Irritant Cathartics.

*Prop.* V.—That the medicine, when in the blood, must permeate the mass of the circulation, so far as may be required to reach the parts on which it tends to act.

That there are two possible exceptions to this rule:—

*a.* The production of sensation or pain at a distant point.

*b.* The production of muscular contraction at a distant point.

*Prop.* VI.—That while in the blood the medicine may undergo changes, which in some cases may, in others may not, affect its influence. That these changes may be—

*a.* Of Combination.

*b.* Of Reconstruction.

*c.* Of Decomposition.

*Prop.* VII.—That a first class of medicines, called Hæmatics, act while in the blood, which they influence. That their action is permanent.

1. That of these some, called Restoratives, act by supplying, or causing to be supplied, a material wanting; and may remain in the blood.

2. That others, called Catalytics, act so as to counteract a morbid material or process; and must pass out of the body.

*Prop.* VIII.—That a second class of medicines, called Neurotics, act by passing from the blood to the nerves or nerve centres, which they influence. That they are transitory in action.

1. That of these some, called stimulants, act so as to exalt nervous force, in general or in particular.

2. That others called Narcotics, act so as first to exalt nervous force, and then to depress it; and have also a special influence on the intellectual part of the brain.

3. That others again, called Sedatives, act so as to depress nervous force, in general or in particular.

*Prop.* IX.—That a third class of medicines, called Astringents, act by passing from the blood to muscular fibre, which they excite to contraction.

*Prop.* X.—That a fourth class of medicines, called Eliminatives, act by passing out the blood through the glands, which they excite to the performance of their functions."

These propositions the author considers the foundation of the Essay, and in the "Superstructure" which he has raised upon them, he has, in our opinion, most fully made good his claim of having "stated about as much of the general principles by which medicines operate as seems to be capable of distinct proof."

The classification adopted, is based upon an exclusion of external or topical agents, among which, we presume, the author would place *Anthelmintics.*

He arranges medicines into "four great groups, the action of each of which is well marked and distinct. The first class acts in the blood; and as a large number of diseases depends on a fault in that fluid, we may by their means be enabled to remedy that fault. They are the most important of all medicines. They are called Hæmatics, or blood medicines. They are used chiefly in chronic and constitutional disorders. But a second class of remedies are temporary in their action. They influence the nervous system, exciting it, depressing it, or otherwise altering its tone. They are chiefly useful in the temporary emergencies of acute disorders. They can seldom effect a permanent cure, unless when the contingency in which they are administered is also of a temporary nature. They are ealled Neurotics, or nerve-medicines. A third set of medicines, less extensive and less important than the others, acts upon muscular fibre, which is caused by them to contract. Involuntary muscular fibre exists in the coats of small blood-vessels, and in the ducts of glands. Thus Astringents, as these agents are called, are able, by contracting muscular fibre, and thus diminishing the calibre of these canals, to arrest hæmorrhage in one case (when a small vessel is ruptured) and to prevent the outpouring of a secretion in another case.

The fourth class is of considerable importance. Some medicines have the power of increasing the secretions which are formed from the blood by various glands at different parts of the body. By their aid we may be enabled to eliminate from the blood a morbid material through the glands; or we may do great good by restoring a secretion when unnaturally suppressed. They are called Eliminatives. Like Hæmatics, their influence is more or less permanent. That of Neurotics and Astringents, particularly the former, is transient."

In allusion to this classification we may be perhaps allowed to say, that it coincides in its great divisions with one adopted in a little work by one of the editors of this journal, with the single exception of the assignment of *Astringents* to an independent class.

Our limits of course forbid a critical analysis of the various subdivisions or orders, into which these principal groups are broken up. Many novel, striking, and ingenious views are presented in the examination of these minor divisions and of some of the more important of the individual articles of the Materia Medica. These theories the author modestly admits to be "the weak points of the Essay."

But in thus boldly venturing upon a path of exploration from which medical inquiry has so long shrunk with unaccountable timidity, Dr. Headland has done good service. For, cértainly, in no branch of medicine is there now the same field for progress as in the advancement of our knowledge of the action of medicines, and of their operation in the cure of disease. Is it not, indeed, surprising, that with the rapid perfection of pathology and diagnosis which has been accomplished within a few years past, so little should have been done to rescue Therapeutics from the doubt and uncertainty with which it has been so long connected!

